# Simple and secure thrombectomy without circulatory arrest for acute pulmonary embolism

**DOI:** 10.1186/s13019-024-02535-y

**Published:** 2024-02-08

**Authors:** Hanae Sasaki, Ryosuke Kowatari, Norihiro Kondo, Masahito Minakawa

**Affiliations:** Department of Thoracic and Cardiovascular Surgery, Hirosaki University School of Medicine, 5 Zaifu-Cho, Hirosaki City, Aomori, 036-8562 Japan

**Keywords:** Massive pulmonary embolism, Thrombectomy, Pulmonary artery

## Abstract

**Background:**

Surgical pulmonary artery thrombectomy is a well-established emergency treatment for massive pulmonary embolism (PE) in which fibrinolysis or thrombolysis are not effective. However, surgery for massive PE that requires peripheral pulmonary artery thrombus removal remains challenging. We established a simple and secure pulmonary artery thrombectomy method using cardiopulmonary bypass and cardiac arrest. In this procedure, the surgical assistant arm, typically used for coronary artery bypass grafting, is used to obtain a feasible working space during thrombectomy.

**Case presentation:**

We present seven consecutive massive PE cases that were treated with the present surgical method and successfully weaned from cardiopulmonary bypass or extracorporeal membrane oxygenation postoperatively.

**Conclusions:**

This procedure can be used to prevent right ventricular failure after surgery as surgeons can remove the peripheral thrombus with clear vision up to the second branch of the pulmonary artery.

## Background

**Fig. 1:**
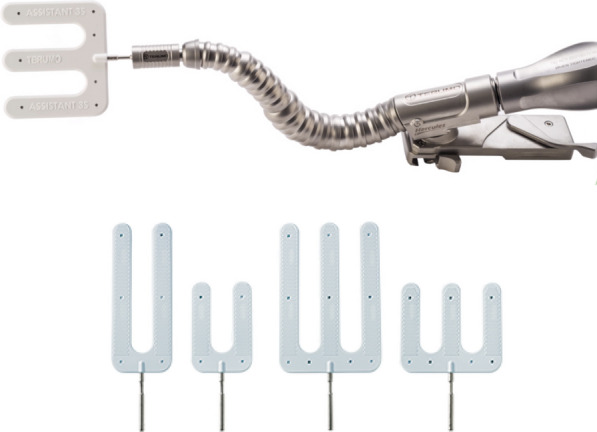
Surgical assistant arm and its attachment

Pulmonary embolism (PE) is a life-threatening condition that often requires emergency surgery. Surgical pulmonary artery (PA) thrombectomy is an established emergency treatment for massive PE [1, 2]. This procedure has facilitated the treatment of most PE cases. However, surgery for severe PE, requiring thrombectomy including peripheral PAs, remains challenging [3]. In such clinical situations, it is difficult to obtain an operative field for direct visualization of the peripheral PA. Recently, we established a simple and secure PA thrombectomy technique utilizing a surgical assistant arm (TERUMO Corporation, Japan), a manual chest retractor attachment used to hold and secure organs such as the heart during surgery, typically used for coronary artery bypass grafting, to obtain a feasible working space (Fig. 1). Herein, we report a case series of acute PE treated using our surgical method involving peripheral PA thrombectomy.

## Case presentation

We applied our technique to seven consecutive patients (one man and six women) between July 2020 and December 2021. The median patient age was 52 (23–77) years. All patients were diagnosed with massive PE confirmed by computed tomography and were unstable circulatory dynamics. Two patients required preoperative circulatory extracorporeal membrane oxygenation (ECMO). The patient demographics are shown in Table 1.

**Table 1:** Patient clinical data

Patient	Age (y), Sex	Massive or sub massive	BMI (kg/m^2^)	Operative time (mins)	CPB time (mins)	Cardiac arrest time (mins)	Preoperative or postoperative ECMO	Intubation time (hours)	ICU stay (days)	Hospital stay (days)	Outcome
1	41, female	Massive	24.2	208	127	65	–	13.5	2	13	Alive
2	69, female	Massive	26.6	265	201	58	Pre	38.5	5	17	Alive
3	23, female	Massive	51.2	263	149	101	–	14	3	16	Alive
4	52, female	Massive	21.3	211	96	53	–	10.5	2	12	Alive
5	52, male	Massive	19.1	320	174	68	–	14	3	14	Alive
6	77, female	Massive	30.1	512	337	43	Pre and post	2688	112	113	Dead
7	49, female	Massive	25.7	272	146	62	Pre and post	95	6	28	Alive

**Fig. 2:**
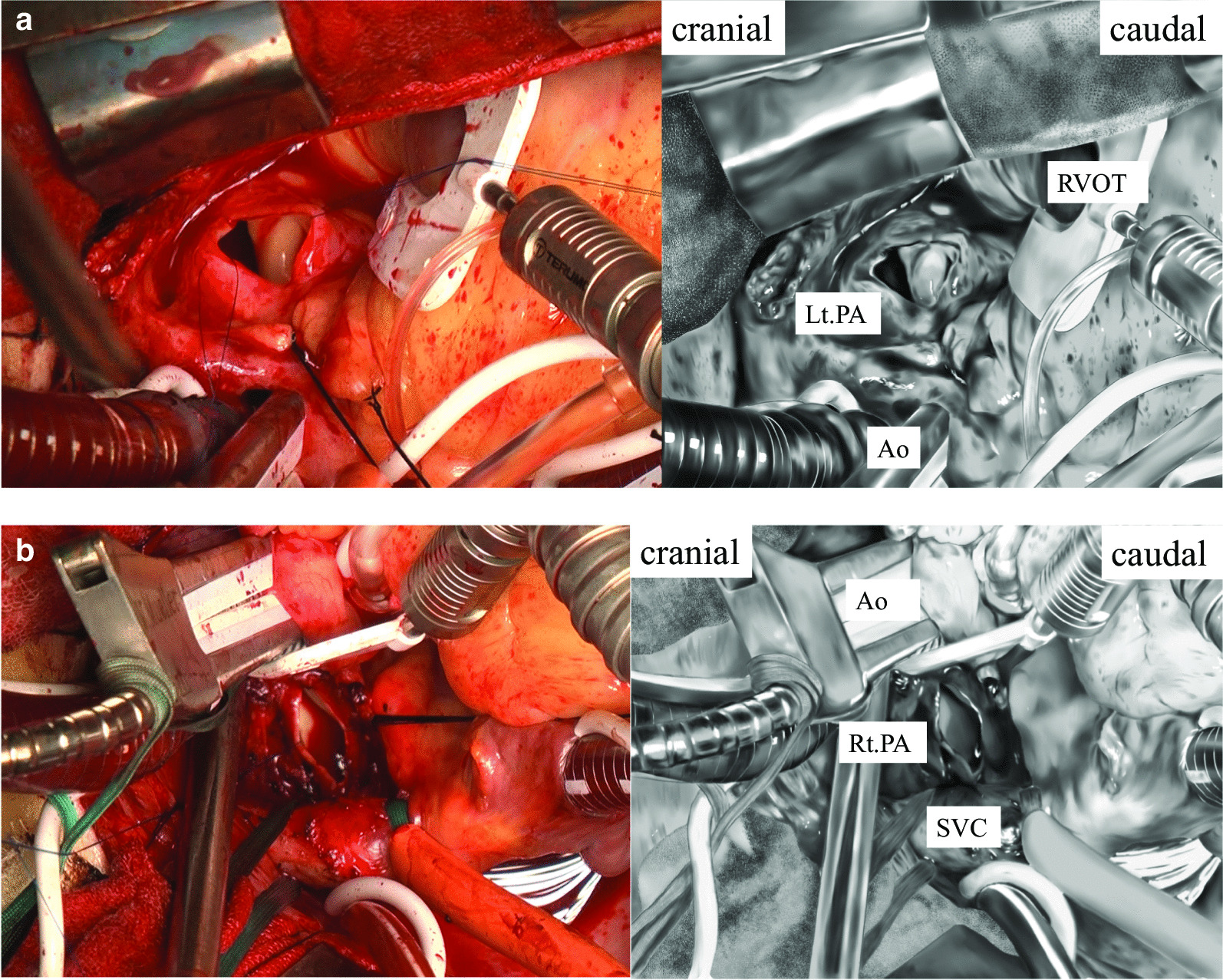
**a** Caudal compression of the right ventricular outflow tract with the surgical assistant arm provides an adequate view of the left pulmonary artery. **b** Pulling up the tourniquet taping the superior vena cava into which the cannula is inserted and compressing the ascending aorta to the left with the arm of the surgical assistant helps obtain a good view of the right pulmonary artery. *PA* pulmonary artery, *RVOT* right ventricle outlet tract, *Ao* aorta, *SVC* superior vena cava

All patients underwent PA thrombectomy using the median sternotomy approach. Cardiopulmonary bypass (CPB) was established with ascending aortic cannulation and bicaval drainage. A left ventricular vent was inserted into the right superior pulmonary vein. Core temperature is controlled above 34 °C. After establishing total bypass, the ascending aorta was cross-clamped, and cardiac arrest was achieved via antegrade cold crystalloid cardioplegia. The surgical assistant arm was then used to compress the right ventricular outflow tract caudally, leading to a sufficient view of the left PA (Fig. 2a). A longitudinal incision was made in the anterior aspect of the main and left PA, and the thrombus was removed. A tourniquet taping the superior vena cava (SVC) with a cannula was pulled up, and the ascending aorta was compressed to the left with a surgical assistant arm (Fig. 2b). A longitudinal incision was made in the anterior aspect of the right PA, enabling us to directly examine the second branch of the right PA after thrombectomy. In contrast to the left side, the thrombus on the right side was torn off several times; however, all thrombi up to the second branch were removed step by step. The surgeon stands to the left of the patient during right-sided thrombectomy. The median operative, CPB, and cardiac ischemic times were 265, 149, and 62 min, respectively. Postoperatively, heparin was used for several days, followed by direct oral anticoagulants or warfarin in all patients. Inferior vena cava filter placement was not performed in all cases.

Five patients who did not require preoperative ECMO were successfully weaned off CPB. Two patients who underwent preoperative veno-arterial ECMO required temporary postoperative veno-arterial ECMO support. One patient developed PE and cardiopulmonary arrest during orthopedic surgery in another hospital and died of multiple-organ failure on postoperative day 112. The median postoperative intubation time, ICU stay, and hospital stay were 14 h, 3 days, and 16 days, respectively. Computed tomography at discharge of the six living patients showed no thrombus within the PA up to the second branch. The patients provided informed consent for the publication of this case series, and the need for ethical approval was waived by our institutional review board.

## Discussion and conclusions

Our experience highlights the reliability of radical thrombectomy in massive PE cases. Thrombectomy for peripheral PE remains a challenge. Thrombectomy in acute PE surgery has often been performed in beating hearts [4, 5]. For massive PE such as in the present cases, surgical thrombectomy is recommended in Class I in the guidelines [6]. Further, thrombolysis is listed as Class I for massive PE. As an institutional policy, we consider surgical thrombectomy to be the first choice for such severe PE cases because of the difficulty in hemostasis after thrombolysis failure. The concern regarding surgery for PE cases is the possibility of residual peripheral PA thrombi. The advantages of pulmonary embolectomy are the reduction of PA pressure immediately after surgery [7] and improvement of right ventricular function after surgery [8], which requires more reliable thrombus removal. Some researchers have recommended gentle thrombectomy with a Fogarty catheter if the thrombus is torn off during thrombectomy to avoid PA injury and the associated pulmonary hemorrhage [9]. However, this approach for peripheral PE is imperfect and can lead to right ventricular failure and/or chronic thromboembolic pulmonary hypertension (CTEPH) [10]. To address this concern, we recently applied the CTEPH technique to treat acute PE. CTEPH surgery aims to reduce postoperative right ventricular pressure by involving a secure endarterectomy of the peripheral PA under circulatory arrest [11, 12]. Moreover, obtaining a clear view of the right PA is challenging in acute PE surgery; mobilizing the SVC and compressing the aorta, as described above, can facilitate the task. Furthermore, the right thrombus of the right PA is more prone to tearing than the left PA, owing to anatomical angulation issues. The technique presented here enables surgeons to obtain clear vision up to the second branch of the PA, making it easier to remove peripheral thrombus. The concept of this technique is similar to that of surgery for CTEPH. The difference with surgery for CTEPH is that this procedure for acute PE does not require circulatory arrest as a fresh thrombus is not strongly adherent to the PA wall. We believe that the cardiac arrest procedure does not adversely affect postoperative cardiac function. All patients without preoperative ECMO support were successfully weaned off CPB. Although one preoperative ECMO case was lost, both preoperative ECMO cases were successfully weaned from ECMO postoperatively. The postoperative course and computed tomography findings indicated that our procedure could accomplish radical thrombectomy of peripheral PE, resulting in low right ventricular pressure and stable hemodynamics postoperatively. Several studies have compared surgical and nonsurgical approaches [7, 13]; however, few have compared the clinical outcomes of each surgical approach. We believe that our technique can be useful for treating severe PE, and more experience should be accumulated.

## Data Availability

The datasets generated and/or analyzed during the current study are available from the corresponding author on reasonable request.
